# Be Straight

**Published:** 1892-12

**Authors:** 


### BE STRAIGHT.

There is every reason for maintaining an erect position in standing or walking, and of being straight even in a sitting position, but no reason can be brought forward why any one should be crooked, except that of natural or accidental deformity. It should be a matter of personal pride with every one to be straight, because by being straight one accepts that great privilege which God has given to humanity only; namely, the power to stand erect, man being furnished with a spine which, properly treated, serves as a column to support the body in an upright position, all the bones, joints, and muscles being arranged to make it possible to maintain this erect posture without fatigue.

A second reason why every one—boy, girl, man or woman—should try by force of the will to be straight, is that grace of figure and movement is impossible with a crooked, stooping body, which not only gives an appearance of ungainliness and awkwardness, but of positive stupidity and slowness of temperament, if not of extreme dullness and laziness amounting to sloth. It is quite possible, indeed it frequently happens, that where there is natural deformity of the spine, the intellect is exceptionally bright and quick, but it is very difficult, as a general thing, to associate a keen intelligence with deformity brought on by carelessness and sloth.

A third and most important reason why people should endeavor to be straight is for health's sake; because a body bent forward by the shoulders and mid-spine, causes compression of the vital organs, the true place to bend the body being the hips. Many persons do not know this, and consequently err ignorantly; instead of keeping the head erect and the shoulders well back and bending forward, when necessary, from the hips, they round their shoulders, crook their spines and crane their heads and necks forward in a most ungainly manner, continuing this habit until by force of habit the position becomes natural.

With the majority of cases these deformities commence in early youth, the child acquiring a habit of lounging at desk or table with the head down, the shoulders rounded, and the spine curved outward, and as a consequence the stomach and abdomen drawn in and compressed. This habit indulged in through the child's growing years, causes ill health and consequent deformity.—*Domestic Journal.*

